# Three-Dimensional Plastic Modeling on Bone Frames for Cost-Effective Neuroanatomy Teaching

**DOI:** 10.7759/cureus.27472

**Published:** 2022-07-30

**Authors:** Manuel de Jesus Encarnacion Ramirez, Renat Nurmukhametov, Gerald Musa, Rossi E Barrientos Castillo, Valerin L. Arno Encarnacion, Jose Antonio Soriano Sanchez, Cesar Augusto Vazquez, Ibrahim E Efe

**Affiliations:** 1 Department of Neurosurgery, People's Friendship University, Moscow, RUS; 2 Department of Biology, Montclair State University, New Jersey, USA; 3 Department of Neurosurgery, ABC Medical Center, Campus Santa Fe, Mexico City, MEX; 4 Department of Urology, Universidad Autonoma de Santo Domingo, Santo Domingo, DOM; 5 Department of Neurological Surgery, Charité - Universitätsmedizin Berlin, Berlin, DEU

**Keywords:** low-cost, training model, education, anatomy, 3d pen, 3d printing

## Abstract

Cadaveric models remain an essential part of medical training across all specialties. Due to their scarcity, high costs, and possible health hazards, there is a need for more accessible and affordable alternatives, especially in low-resource settings. We introduce cost-effective and easily replicable three-dimensional (3D) printed models to help democratize access to hands-on neuroanatomy education.

Silicone-based glue is applied on the surface of a 3D-printed or cadaveric bone frame. Using plastiline on a 3 mm 3D acrylonitrile butadiene styrene pen, the desired anatomical structure is printed on the bone frame. A heat gun is used to smoothen the plastic edges. The structure can then be painted according to its appearance in the real anatomy.

Using this technique, we successfully generated a variety of anatomical models to study the cerebrovascular anatomy, the course of the cranial nerves in relation to the skull base, and extracranial structures including the spine.

Procurement and conservation of cadaveric specimens can be cumbersome. Our model may be an affordable and easily replicable approach to bridging the gap in anatomy education between low- and high-resource facilities.

## Introduction

Anatomy is an invaluable part of every medical training program. Textbooks offer a two-dimensional (2D) perspective on anatomy only. A thorough understanding of human gross anatomy requires many hours of training with anatomically accurate three-dimensional (3D) specimens. In neurosurgical training, spatial 3D visualization is of utmost importance. It allows the trainee to appreciate various anatomical relations and interactions and how they translate to, i.e. surgical corridors and focal neurologic deficits. This is mainly achieved through tangible 3D models [1]. Cadavers remain the gold standard for hands-on neuroanatomy education. Unfortunately, not all centers can procure and preserve cadavers. In addition, prolonged exposure to formalin may be detrimental to one’s health [2]. In this regard, the authors present an affordable, safe, and easily replicable 3D anatomy teaching model to enhance the availability and accessibility of hands-on neuroanatomy education globally.

## Technical report

These models rely on bony frames that can either be 3D-printed or derived from preserved human bones. Here, both 3D-printed and real human bones were used. The area of the bone onto which the anatomical structure is printed is cleaned thoroughly. A silicone-based glue (Henkel Moment Universal Classic, Henkel AG & Co. KGaA, Dusseldorf, Germany) is then applied onto the cleaned and dried surface. Using plastiline material on a 3 mm 3D acrylonitrile butadiene styrene pen, the structure is manually printed on the bony frame. The models shown here were created with the aid of the INVENTO 3D Pen-2. We relied on conventional anatomical atlases and digital 3D models to accurately reproduce the human anatomy. Yet, accuracy depends on the individual user’s spatial understanding and ability to correctly handle the 3D pen. The edges of the anatomical structure may be rough and uneven. For this reason, a heat gun is used to smoothen irregularities and allows for the molding of areas that need additional correction. The final product is painted using acrylic paint using colors that resemble the real anatomy. Varnish is applied to achieve a shiny texture. 

**Figure 1:**
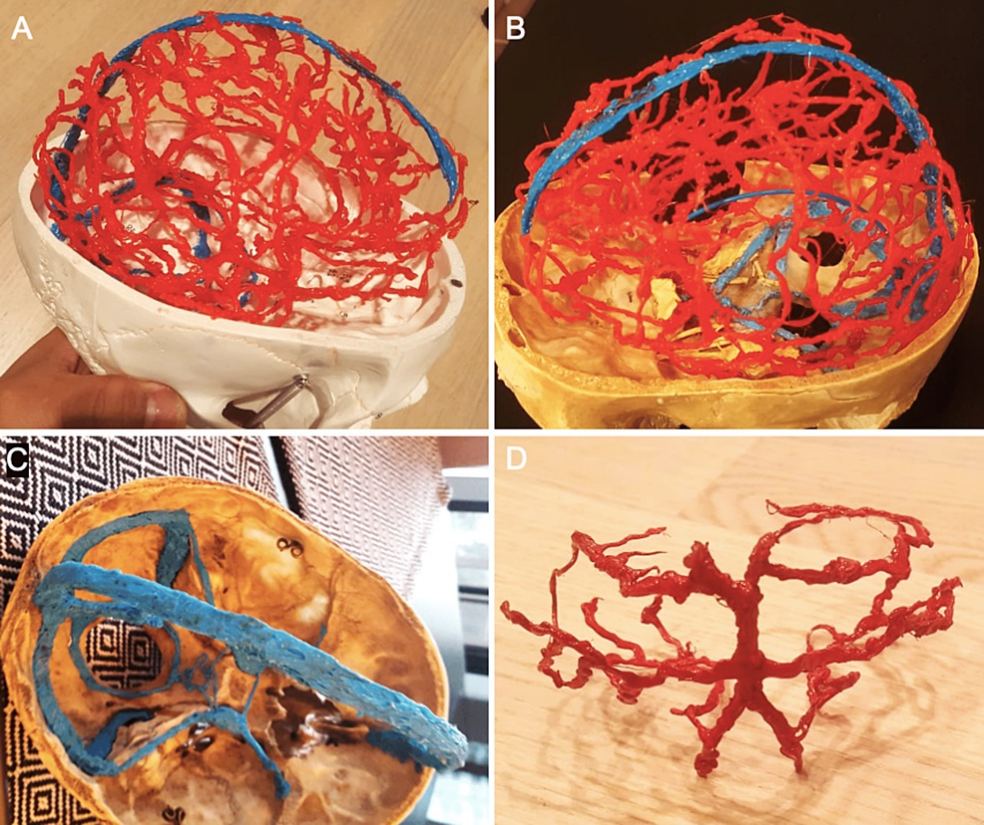
(A, B) The arterial (red) and venous (blue) vasculature of the intracranial cavity has been printed onto a skull base model illustrating the three-dimensional (3D) relationships between vascular and bony structures. (C) The dural venous sinuses have been 3D-printed onto the skull base in blue. (D) 3D-printed objects can be easily detached from the underlying bone frame causing no damage to the bone or the 3D-printed structure. The posterior arterial circulation was initially printed on a cadaveric skull base and then detached.

Using a low-cost 3D acrylonitrile butadiene styrene pen in combination with 3D-printed or cadaveric bone frames, we were able to generate a variety of anatomical models. To study the cerebrovascular anatomy, the major arterial vessels and venous sinuses of the intracranial cavity were 3D-printed manually onto an original size skull base model (Figure 1A-1C). This model was completed in approximately 5.5 hours. The 3D-printed structures could be easily detached from the bony surface without causing any damage to the bone frame or the 3D-printed object (Figure 1D).

**Figure 2:**
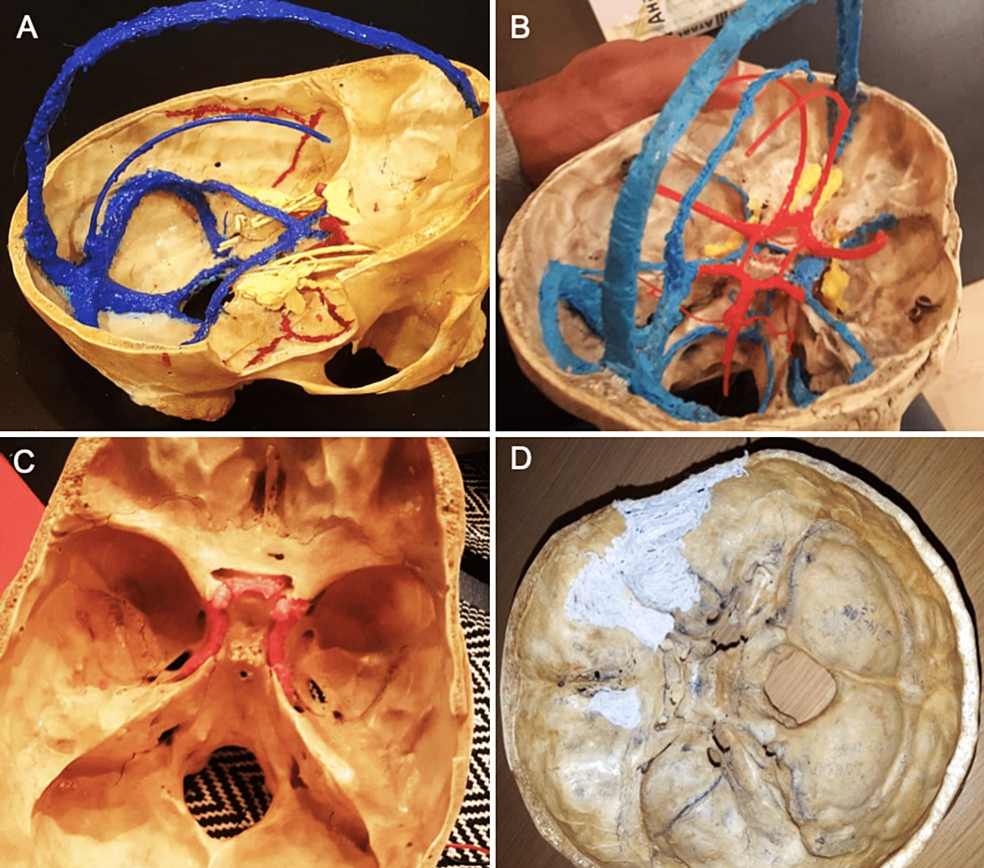
(A, B) The dural venous sinuses (blue), the circle of Willis (red), and cranial nerves III, IV, and V (yellow) have been three-dimensional (3D)-printed to demonstrate their course in relation to the skull base. (C) The course of the internal carotid artery (red) is easily traceable. (D) 3D printing can be used to preserve or reconstruct defective bone parts and anatomical models.

For a better understanding of the relationship between the cranial nerves and the skull base, we created two models involving the oculomotor nerve, trochlear nerve, and trigeminal nerve (yellow) along with the dural venous sinuses (blue) and the circle of Willis (Figure 2A, 2B). Both models were completed in approximately 3 hours each. In another model, we were able to reproduce the course of the internal carotid artery from the carotid canal to the anterior clinoid process in roughly 25 minutes (Figure 2C). Further, we found our technique to be helpful in repairing and preserving bony structures. A skull base with a sphenoid bone fracture could be restored through plastiline printing and remodeling with a heat gun (Figure 2D). 

**Figure 3:**
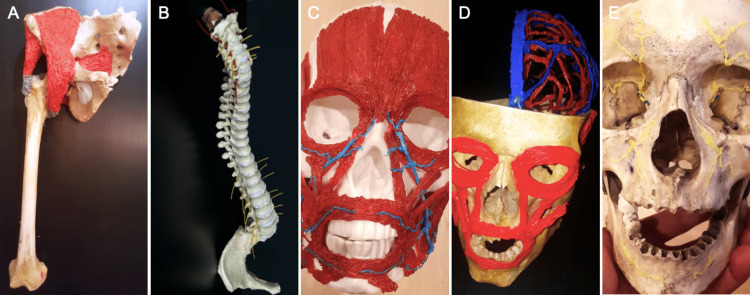
(A) The hip muscles have been drawn onto a real femur and pelvis bone to show the attachment of the major muscles responsible for hip movement. (B) Three-dimensional (3D)-printed spinal nerves can be studied in relation to various bony and vascular structures. (C) Muscles of facial expression and mastication are printed onto a skull model. The branches of the facial vein can be seen in blue. (D) Both facial muscles and the intracranial vasculature of the left hemisphere can be seen. (E) The course of the trigeminal nerve branches has been drawn in yellow.

We then explored the usefulness of our technique for extracranial anatomy and found it to be equally feasible and effective for several structures including facial muscles, trigeminal nerve branches, hip muscles, and neurovascular structures of the vertebral column (Figure 3A-3E). It should be noted, however, that these models may not accurately depict the course of the muscle fibers. 

## Discussion

Anatomy education for medical students and surgical residents relies primarily on conventional 2D sources, mostly in the form of lectures, digital presentations, and videos [3,4]. Spatial understanding of anatomy, however, is crucial for students and even more so for residents in neurosurgery and other surgical specialties [5]. Three-dimensional printing has emerged as an effective modality for preoperative surgical planning [4,6]. The incorporation of 3D printing in the training of students and residents has begun to change the landscape of anatomy education. These models allow the student to appreciate the real-life-like anatomy that is otherwise compromised in cadavers through the preservation process [7-9]. Not only neurosurgeons but also other surgical disciplines as well as veterinary specialists have adopted this technology to create organ models [4,6,10,11]. Three-dimensional printed models can further be used to improve manual dexterity and simulate procedures [6].

This technology may help overcome the major limitations of cadaver-based anatomy teaching. The difficulty of obtaining and maintaining corpses has been considered a major challenge, particularly in low-resource settings. Meanwhile, 3D printing technologies gain more widespread popularity due to decreasing costs and more user-friendly handling [1]. The ability to combine multiple materials within a single 3D-printed model allows for an accurate and patient-specific reconstruction of the pathology and provides an opportunity to simulate and compare surgical approaches. We used the INVENTO 3D Pen-2 which we purchased for $28.82. However, most commercially available low-budget 3D pens are capable of achieving similar results. The 3D-printed objects can be easily detached from the bony surface without causing damage to any structure. They can later be placed back onto the frame when needed. In this way, a single skull base frame can be quickly customized to accommodate various 3D-printed structures depending on the needs of the student or the surgeon. 

Besides neuroanatomy teaching, we identified its use in restoring fractured bones. It could further be used for patient education and consultation. For this purpose, one can reproduce a patient’s pathology by modeling an aneurysm or skull base tumor. Finally, the production of the models helps the creator consolidate his or her anatomical knowledge. Thus, our method may provide an educational benefit for both the models’ user and creator. 

Limitations

A major limitation of our method is that achieving high accuracy requires multiple hours of experience. The models shown in this study have been created after a total of 8 hours of hands-on practice over the course of two weeks. The cerebral vasculature model shown in Figures 1A, 1B was completed in close to 6 working hours. Regardless of experience, our models remain prone to human error. Hence, the production depends on the use of conventional anatomical atlases as templates to frequently compare and correct the printed object if necessary. Both the anatomical accuracy and educational benefit of our models remain to be quantified. Although this technique may not be sufficiently accurate for preoperative planning, we believe it holds potential for medical student and resident education as well as informed consent in doctor-patient communication.

## Conclusions

Access to cadaver-based anatomy education is limited due to the paucity of specimens, high procurement costs, and strict regulations. Further, the preservation of cadavers depends on adequate facilities and exposure to potentially hazardous formalin. Our technique is a simple and affordable approach to generating easily replicable anatomical 3D models. Three-dimensional printing technologies can help narrow the gap in neuroanatomy education between low- and high-resource facilities.

## References

[1] Tabernero Rico RD, Juanes Méndez JA, Prats Galino A (2017). New generation of three-dimensional tools to learn anatomy. J Med Syst.

[2] Hauptmann M, Stewart PA, Lubin JH (2009). Mortality from lymphohematopoietic malignancies and brain cancer among embalmers exposed to formaldehyde. J Natl Cancer Inst.

[3] Kurt E, Yurdakul SE, Ataç A (2013). An overview of the technologies used for anatomy education in terms of medical history. Procedia Soc Behav Sci.

[4] ten Brinke B, Klitsie PJ, Timman R, Busschbach JJ, Lange JF, Kleinrensink GJ (2014). Anatomy education and classroom versus laparoscopic dissection-based training: a randomized study at one medical school. Acad Med.

[5] Falah J, Khan S, Alfalah T, Alfalah SFM, Chan W, Harrison DK, Charissis V (2014). Virtual reality medical training system for anatomy education. 2014 Science and Information Conference.

[6] Mooney MA, Cavallo C, Zhou JJ (2020). Three-dimensional printed models for lateral skull base surgical training: anatomy and simulation of the transtemporal approaches. Oper Neurosurg (Hagerstown).

[7] Hopkins R, Regehr G, Wilson TD (2011). Exploring the changing learning environment of the gross anatomy lab. Acad Med.

[8] Cheng X, Wang L, Guo K, Liu S, Li F, Chu G, Zhou LH (2011). Postgraduate fellows as teaching assistants in human anatomy: an experimental teaching model at a Chinese research university. Anat Sci Educ.

[9] Chan LK, Cheng MM (2011). An analysis of the educational value of low-fidelity anatomy models as external representations. Anat Sci Educ.

[10] Hackmann CH, dos-Reis D de AL, de-Assis-Neto AC (2019). Digital revolution in veterinary anatomy: confection of anatomical models of canine stomach by scanning and three-dimensional printing (3D). Int J Morphol.

[11] Sun Z, Squelch A (2015). 3D printed models of complex anatomy in cardiovascular disease. Heart Res Open J.

